# An Enhanced Neural Network Algorithm with Quasi-Oppositional-Based and Chaotic Sine-Cosine Learning Strategies

**DOI:** 10.3390/e25091255

**Published:** 2023-08-24

**Authors:** Xuan Xiong, Shaobo Li, Fengbin Wu

**Affiliations:** 1 State Key Laboratory of Public Big Data, College of Computer Science and Technology, Guizhou University, Guiyang 550025, China; gs.xxiong22@gzu.edu.cn; 2 State Key Laboratory of Public Big Data, Guizhou University, Guiyang 550025, China

**Keywords:** neural network algorithm, quasi-oppositional-based learning, complex optimization, chaotic mapping, sine-cosine learning strategy, a new strategy, CEC 2017

## Abstract

Global optimization problems have been a research topic of great interest in various engineering applications among which neural network algorithm (NNA) is one of the most widely used methods. However, it is inevitable for neural network algorithms to plunge into poor local optima and convergence when tackling complex optimization problems. To overcome these problems, an improved neural network algorithm with quasi-oppositional-based and chaotic sine-cosine learning strategies is proposed, that speeds up convergence and avoids trapping in a local optimum. Firstly, quasi-oppositional-based learning facilitated the exploration and exploitation of the search space by the improved algorithm. Meanwhile, a new logistic chaotic sine-cosine learning strategy by integrating the logistic chaotic mapping and sine-cosine strategy enhances the ability that jumps out of the local optimum. Moreover, a dynamic tuning factor of piecewise linear chaotic mapping is utilized for the adjustment of the exploration space to improve the convergence performance. Finally, the validity and applicability of the proposed improved algorithm are evaluated by the challenging CEC 2017 function and three engineering optimization problems. The experimental comparative results of average, standard deviation, and Wilcoxon rank-sum tests reveal that the presented algorithm has excellent global optimality and convergence speed for most functions and engineering problems.

## 1. Introduction

In contemporary practical applications, it is significant to imperative tackle a wide variety of optimization problems. These encompass the optimization of route planning [1,2], production scheduling [3,4], energy system [5], nonlinear programming [6], supply chain [7], facility layout [8], medical registration [9], and unmanned system [10], among others. These projects typically involve an enormous amount of information and constraints where conventional algorithms would struggle to find an optimal solution within a reasonable timeframe. Consequently, investigating efficient approaches to these intricate optimization processes has become an extremely challenging research domain. After relentless efforts, there are numerous optimization methods exploited by researchers, commonly employing deterministic and meta-heuristic approaches over intricate optimization issues.

Deterministic methods can be described as problem-solving approaches that rely on rigorous logic and mathematical models, effectively utilizing extensive gradient information to search for optimal or near-optimal solutions [11]. However, the strong dependence on the initial starting point makes it easy to produce identical results. In the real world, optimization problems are often highly intricate and exhibit nonlinear characteristics [12], which frequently involve multiple local optima within the objective function. Consequently, deterministic methods often encounter difficulties in escaping local minima when dealing with complex optimization problems [13,14]. Instead, metaheuristics are inspired by phenomena observed in nature and simulate these phenomena to efficiently optimize and solve problems without relying on complex gradient information and mathematical principles thereby better exploring optimal solutions [15,16,17]. For instance, the grey wolf optimization (GWO) [18] replicates the social behavior of grey wolves during the search for optimization; the artificial immune algorithm (AIA) [19] mimics the evolutionary process of the human immune system to adaptively adjust the solution quality; the ant colony optimization (ACO) [20] emulates the pheromone-based foraging behavior of ants. It is noteworthy that the parameters of metaheuristic algorithms can be classified into two categories [21]: common parameters and special parameters. Common parameters encompass the foundational principles that govern the behavior of an algorithm, such as population size and termination criteria. On the other hand, specific parameters are tailored to the unique characteristics of individual algorithms. For instance, in simulated annealing (SA) [22] configuring the initial temperature and cooling rate is crucial for achieving optimal outcomes. Given the sensitivity of the algorithms for input data, any improper tuning of specific parameters may contribute to an augmented computational effort or the conundrum of local optimality when treating varying sorts of projects.

It is for heuristic algorithms featuring non-specific parameters that have gained immense relevance. Neural network algorithm (NNA) [23], which draws inspiration from artificial neural networks and biological nervous systems, emerged in 2018 as a promising method towards achieving globally optimal solutions. Additionally, a distinguishing trait of NNA from many famous heuristic algorithms is that it relies only on common parameters; hence, no extra parameters are required. This universality dramatically enhances its superior adaptability across a range of engineering applications. Nevertheless, NNA is confronted with two notable constraints: susceptibility to local optima and sluggish convergence speed. Therefore, a lot of improved optimization algorithms based on the scientific method have been offered to ameliorate the defects of NNA. For example, the competitive learning chaos neural network algorithm (CCLNNA) [24] is proposed by integrating NNA with competitive mechanisms and chaotic mapping; an effective hybrid algorithm TLNNA based on TLBO algorithm and NNA is proposed [25]; the gray wolf optimization neural network algorithm (GNNA) was created by combining GWO with NNA [26]; and the dropout strategy in the neural network was introduced and the elite selection strategy was proposed as a neural network algorithm with dropout using elite selection [27]. Moreover, by the no free lunch theorem [28], no one algorithm can be applied to all optimization questions. Thereby, it is fundamental for the ongoing refinement of existing to develop novel algorithms along with the integration of multiple algorithms for better results under practical applications. In this paper, the quasi-oppositional and chaotic sine-cosine neural network algorithm to boost the global search capability and refine the convergence performance of NNA is proposed. The main contributions of this work are listed below:

- To maintain the QOCSCNNA diversity of populations, a quasi-oppositional-based learning (QOBL) [29] is introduced, where quasi-opposite populations are randomly generated between the centers of solution space and opposite space, which contributes to better balance exploration and exploitation that make these populations closer to the most optimal ones more likely.
- By integrating logistic chaotic mapping [30] and sine-cosine strategy [31], a new logistic chaotic sine-cosine learning strategy (LCSC) is proposed that helps to escape from local optimum in the bias strategy phase.
- To improve the QOCSCNNA convergence performance, a dynamic tuning factor of piecewise linear chaotic mapping [32] is employed to adjust the chances of operation for the bias and transfer operators.
- The optimization performance of QOCSCNNA was verified through 29 numerical optimization problems based on the CEC 2017 test suite [33], as well as two real-world engineering constraint problems.

The remainder of this paper follows the following structure: a brief introduction of the original NNA is given in Section 2. Section 3 describes the proposed QOCSCNNA in detail. Section 4 validates the performance of the QOCSCNNA as well as explores the application of the QOCSCNNA to real-world engineering design problems using the CEC 2017 test suite. Finally, the main conclusions of this paper are summarized in Section 5 and further research directions are proposed.

## 2. NNA

**Figure 1:**
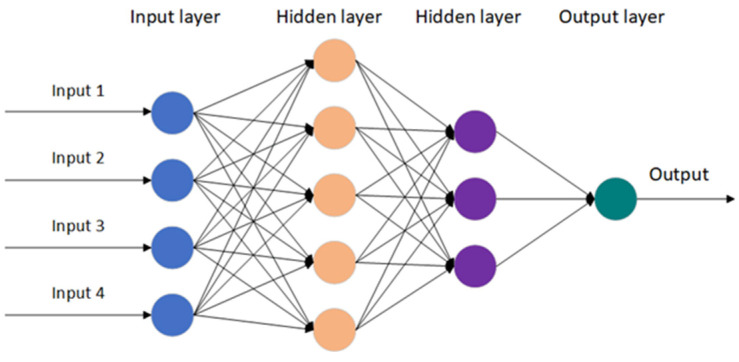
Structure of an artificial neural network.

Artificial neural networks (ANNs) are mathematical models that are based on the principles of biological neural networks, aiming to simulate the mechanisms of information processing in the human brain. ANNs are used for prediction primarily by receiving input data and output data which infer the relationship between these. The input data for ANN are typically obtained through experiments, computations, and other means, and the weights are iteratively adjusted to minimize the error between the predicted solution and the target solution, as shown in Figure 1. However, it might sometimes be unknown what the target solution is. Aiming to solve in this way, the authors of NNA treat the current best solution as the target solution and keep adjusting the weights of each neuron to achieve it. The NNA is a population-based evolutionary algorithm, which involves initializing the population, updating the weight matrices, and setting bias operators, and transferring operators.

### 2.1. Initial Population

In the NNA algorithm, the population is updated using a neural network model-like approach. In the search space, the initial population $X^{r}=[x_{1}^{r},x_{2}^{r},\ldots ,x_{N}^{r}]$ is updated through the weight matrix $W^{r}=[w_{1}^{r},w_{2}^{r},\ldots ,w_{N}^{r}]$, for any generation (*r*). Here, $x_{i}^{r}$ represents the $i^{th}$ individual vector and $w_{i}^{r}$ represents the $i^{th}$ weight vector, both with *D* dimensions. Thus, $x_{i}^{r}=[x_{i,1}^{r},x_{i.2}^{r},\ldots ,x_{i,D}^{r}]$ and $w_{i}^{r}=[w_{i,1}^{r},w_{i.2}^{r},\ldots ,w_{i,D}^{r}]$, where $i=1,2,\ldots ,N_{p}$.

It is desirable to impose constraints on the weights associated with new model solutions so that significant biases are prevented in the generation and transmission of these solutions. In this way, NNA was equipped to regulate its behavior through subtle deviations. After initializing the weights, the one corresponding to the desired solution ($X_{target}$), i.e., the target weight ($W_{target}$), that is chosen from the weight matrix *W*. Therefore, the summation of the weight matrix must adhere to the following conditions:

(1) $$\sum_{j=1}^{N}w_{i,j}^{r}=1,\;\;i=1,2,\ldots ,N_{p}$$

where

(2) $$w_{i,j}\in U\left[0,1\right],\;\;i,j=1,2,\ldots ,N_{p}$$

In addition, the formula of generating a new population at the ${\left(r+1\right)}^{th}$ iteration can be expressed by:

(3) $$x_{i,new}^{r+1}=\sum_{i=1}^{N}w_{i,j}^{r}\times x_{i}^{r},\;\;i=1,2,\ldots ,N,\;\;j=1,2,\ldots ,N_{p}$$

(4) $$x_{i}^{r+1}=x_{i}^{r}+x_{i,new}^{r+1},\;i=1,2,\ldots N_{p}$$

where $N_{p}$ is the population size, *r* is the current number of iterations, and$\;x_{i,new}^{r}$ is the weighted solution of the $i^{th}$ individual at time *r*.

### 2.2. Update Weight Matrix

The weight matrix is then adjusted based on the desired target weight $\left(W_{target}\right)$ using the following formula:

(5) $$w_{i}^{r+1}=\left|w_{i}^{r}+2\times rand(0,1)\times \left(w_{target}^{r}-w_{i}^{r}\right)\right|,\;\;i=1,2,\ldots ,N_{p}$$

where $w_{target}^{r}$ is the vector of optimal target weights obtained in each iteration.

### 2.3. Bias Operator

To enhance the global search capability of NNA, a bias operator has been incorporated to fine-tune the probabilities of pattern solutions generated using the new population and updated weight matrices. A correction factor *β* is utilized to precisely define the probability of the adjusted pattern solution. Initially, *β* is initialized to 1 and progressively decreased in each iteration. The update process can be outlined as follows:

(6) $$\beta^{r+1}=\beta^{r}\times 0.99,\;\;r=1,2,\ldots ,T_{max}$$

The bias operator encompasses two components: the bias population and the bias weight matrix. To begin, a random number $N_{P}$ and a set *P* are generated, where $N_{P}$ is *D* multiplied by $\beta^{r}$. Let $L=(l_{1},l_{2},\ldots ,l_{D})$ and $U=u_{1},u_{2},\ldots ,u_{D})$ be the lower and upper limits of the variables Additionally, *P* denotes a set of $N_{P}$ integers that are randomly selected from the range of 0 to *D*. Consequently, the definition of the bias population can be formulated as follows:

(7) $$x_{i,P(s)}^{r}{=l}_{P(s)}+\left(u_{P\left(s\right)}-l_{P\left(s\right)}\right)\times \alpha_{1},\;\;s=1,2,\ldots ,N_{P}$$

where $\alpha_{1}$ is a random number between 0 and 1 that obeys a uniform distribution. The bias weight matrix also involves two variables: a random number $P_{w}$, a stochastic number determined by the formula $N\times \beta^{r}$, and *Q*, a set of $P_{w}$ integers randomly chosen between 0 and *N*. Therefore, the scientific representation for defining the bias weight matrix can be formulated as follows:

(8) $$w_{i,Q\left(t\right)}^{r}=\alpha_{2},t=1,2,\ldots ,P_{w}$$

where $\alpha_{2}$ is a random number between 0 and 1, following a uniform distribution. 

### 2.4. Transfer Operator

There is an introduced transfer function operator (TF) that transfers the new mode solution at the current position to a new position in the search space proximal to the target solution ($x_{target}^{r}$). This operator can be denoted as:

(9) $$x_{i}^{r+1}=x_{i}^{r}+2\times \alpha_{3}\times {(x}_{target}^{r}-x_{i}^{r}),\;\;i=1,2,\ldots ,N_{P}$$

where $\alpha_{3}$ is a random number between 0 and 1 that follows a uniform distribution. Based on the above statements, the overall NNA framework can be seen in the pseudocode in Algorithm 1.
**Algorithm 1:** The pseudocode of the NNA algorithmInitialize the population $X^{r}$ and the weight matrix $W^{r}$.Calculate the fitness value of each solution and then set $X_{target}$ and $W_{target}$for $i=1:N_{P}$  Generate the new solution $x_{i}^{r}$ by Equation (3) and new weight matrix $w_{i}^{r}$ by Equation (5)  if $\beta^{r}\geq rand$  Perform the bias operator for $x_{i}^{r+1}$ by Equation (7) and the weight matrix $w_{i}^{r+1}$ by Equation (8)  else  Perform the transfer function operator for $x_{i}^{r}$ via Equation (9)  end ifend forGenerate the new modification factor $\beta^{r+1}$ by Equation (6)Calculate the fitness value of each solution and find the optimal solution and the optimal weightUntil(stop condition = false)Post process results and visualization

## 3. Quasi-Oppositional-Based Chaotic Sine-Cosine Neural Network Algorithm

### 3.1. Quasi-Oppositional-Based Learning Strategy

Opposites-based learning (OBL) theory [34] has been proposed by Tizhoosh to synthesize the selection of existing solutions and their opposites to improve the quality of candidate solutions. The OBL strategy can provide more accurate candidate solutions. Moreover, the OBL theory evolved into quasi-oppositional-based learning (QOBL) approaches, which show a higher probability of approaching the unknown optimal solution compared to the candidate solutions generated by OBL in terms of achieving the global optimum [29]. To enhance the quality and convergence speed of the solutions, researchers integrated QOBL into metaheuristic methods.

**Figure 2:**
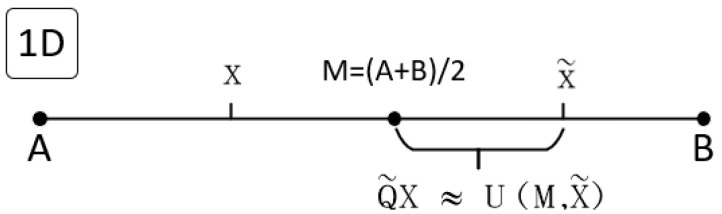
One-dimensional space opposites points and quasi-opposite points.

The opposite point is the symmetric point of a given point concerning the center point in the solution space. Figure 2 shows the positions of the current point, the opposite point $\tilde{X}$, and the quasi-opposite $\tilde{QX}$ within the one-dimensional space [A, B]. IF ${X=(x}_{1},x_{2},\;\ldots ,x_{n}$) represents a point in an n-dimensional space, where each coordinate $x_{i}\in [a_{i},b_{i}]$ for *i* = 1, 2, …, *n*. The opposite point $\tilde{X}=(\tilde{x_{1},}\tilde{x_{2},\;}\ldots \tilde{x_{n}})$ corresponding to the generated X is as follows:

(10) $$\tilde{x_{i}}=a_{i}+b_{i}-x_{i}$$

Furthermore, the quasi-opposite point $\tilde{QX}=(\tilde{{qx}_{1},}\tilde{{qx}_{2},\;}\ldots \tilde{qx_{n}})$ is randomly generated between the inverse point and the center point M=(A+B)/2 of the solution space. The quasi-opposite point $\tilde{QX}$ of $\tilde{X}$ can be generated as follows [29]:

(11) $$\tilde{{qx}_{i}}=\left\{\begin{array}{c} m_{i}+\tilde{(x_{i}}-m_{i})\times k,\;m_{i}<\tilde{x_{i}}\\ \tilde{x_{i}}+(m_{i}-\tilde{x_{i}})\times k,\;m_{i}>\tilde{x_{i}} \end{array}\right.$$

where *k* is a uniformly distributed random number between 0 and 1.

In this study, QOBL performs the initialization and generation of jumps for QOCSCNNA. The initialization phase through which randomly generated initial populations of quasi-opposite populations is created. The ridiculously generated initial population is taken to define the optimal solution of the inception phase; the generation jumping phase drives the algorithm jumps during the selection process to the solution with a better fitness function value. In this process, a greedy strategy is used to decide whether to keep the current solution or leap to a quasi-opposite solution. The pseudocode for the QOBL strategy is presented in Algorithm 2.
**Algorithm 2:** QOBL Strategy
for $i=1:\;N_{P}$
 for *j* = 1: *N*  
$\tilde{x_{i}}=\alpha_{i}+\gamma_{i}-x_{i}$
  
$m_{i}=(\alpha_{i}+\gamma_{i})/2$
    If $m_{i}<\tilde{x_{i}}$
    
$\tilde{{qx}_{i}}=m_{i}+\tilde{(x_{i}}-m_{i})\times k$
    else     
$\tilde{{qx}_{i}}=\tilde{x_{i}}+{(m}_{i}-\tilde{x_{i}})\times k$
    end if end forend for

### 3.2. Chaotic Sine-Cosine Learning Strategy

#### 3.2.1. Sine-Cosine Learning Strategy

For the performance improvement of meta-heuristic algorithms, Mirjalili introduced the sine-cosine learning strategy (SCLS) in his research [31]. It is the core idea of this strategy that the current solution is updated using the sine and cosine functions which effectively refrain the algorithm from falling into a local optimum. The definition of the algorithm is given below [31]:

(12) $$x_{i,j}^{r+1}=\left\{\begin{array}{c} x_{i,j}^{r}+u_{1}\times \sin\left(u_{2}\right)\times \left|u_{3}\times x_{target}^{r}-x_{i,j}^{r}\right|,\;u_{4}<0.5\\ x_{i,j}^{r}+u_{1}\times \cos(u_{2})\times \left|u_{3}\times x_{target}^{r}-x_{i,j}^{r}\right|,\;u_{4}\geq 0.5 \end{array}\right.$$

where *r* is the current iteration number; $x_{i,j}^{r}$ is the position of the $i^{th}$ individual in the $j^{th}$ dimension in the r iteration; and $x_{target}^{r}$ is the optimal solution of the previous generation. $u_{2}$ is a range greater than 0 and less than $\sqrt{2}$ as the radius of the circles. $u_{3}$ is set to be a random number between 0 and 2, to control the distance of the optimal solution and maintain the diversity of the population. The value of $u_{4}$ is a random number between 0 and 1. *u*_1_ is the cosine amplitude adjustment factor, set as follows:

(13) $$u_{1}=\frac{r}{R}$$

where *R* is the maximum number of iterations.

#### 3.2.2. Logistic Chaos Mapping

The exploratory potential of chaos optimization algorithms can be further enhanced by leveraging the traversing traits and stochastic attributes of chaotic variables to optimize the diversity within the population [35]. In this work, the well-known logistic chaos mapping (LCM) was chosen to generate chaotic candidate solutions. Logistic mapping is formulated as follows [30]:

(14) $$c_{z+1}=p\times c_{z}\times (1-c_{z})$$

where $c_{z}\in$ (0, 1) ∀ *z*∈{0, 1, …$N_{P}$} and *p* = 3.8.

Based on the candidate solutions generated by the logistic chaos, the generation is as follows:

(15) $$\delta_{z}{=l}_{z}+\left(u_{z}-l_{z}\right)\times c_{z}$$

In biased operators, a novel strategy, i.e., logistic chaotic sine-cosine learning strategy (LCSC), is generated by integrating the LCM with the SCLS to align the candidate solutions to be that more chaotic to explore the design space. This mechanism serves as a preventive measure against premature convergence in subsequent iterations. The new solution is generated as follows:

(16) $$x_{i,j}^{r+1}=\left\{\;\begin{array}{c} \delta_{z}+u_{1}\times \sin\left(u_{2}\right)\times \left|u_{3}\times c_{z}-x_{i,j}^{r}\right|,\;{\;u}_{4}<0.5\\ \delta_{z}+u_{1}\times \cos\left(u_{2}\right)\times \left|u_{3}\times c_{z}-x_{i,j}^{r}\right|,\;u_{4}\geq 0.5 \end{array}\right.$$

where *r* is the current iteration number; $x_{i,j}^{r}$ is the position of the $i^{th}$ individual in the $r^{th}$ iteration. $u_{1}$ is the positive cosine amplitude adjustment factor, defined as shown in Equation (12). $u_{2}$ is set to be more than 0 and smaller than a circle with a radius of $\sqrt{2}$, $u_{3}$ is set to be a random number between 0 and 2, and $u_{4}$ is set to be a random number between 0 and 1. The pseudocode of the bias operator changed by the CSCL is given in Algorithm 3.
**Algorithm 3:** The Bias Operator$P_{n}$ signifies the number of biased variables in the population of the new pattern solution$P_{w}$ signifies the number of biased variables in the updated weight matrixfor i = 1: $N_{P}$
 if
rand≤β
  %Bias for new pattern solution %  $P_{n}=round(N\times \beta )$
  Update the chaotic sequence $\delta_{z}$ using Equation (14)  for j = 1: $P_{n}$
    Update the new pattern solution $x_{i,Integer\;rand[0,N]}^{r+1}$ by Equation (16)  end for  %Bias for updated weight %  
$P_{w}=round(N_{P}\times \beta )$
  for j = 1: $j=1:P_{w}$
   Update the weight $w_{j,Integer\;rand[0,N_{P}]}^{r}$ by Equation (8)  end for end ifend for

### 3.3. The Dynamic Tuning Factor

Since the bias operator decreases as the number of iterations increases, a piecewise linear chaotic map (PWLCM) [32] is introduced, for which the chances of running different learning strategies are dynamically tuned to help QOCSCNNA converge faster as more iterations are added. As well, the definition of PWLCM is denoted in Equation (17):

(17) $$Z_{r+1}=\left\{\begin{array}{c} \;Z_{r}/k\;,\;{\;Z}_{r}\epsilon (0,k)\;\\ \left(1-Z_{r}\right)/\left(1-k\right),\;{\;Z}_{r}\epsilon [k,1) \end{array}\right.$$

where *r* represents the function mapping value for the $r^{th}$ iteration; *k* is a control parameter, with k between 0 and 1.

**Figure 3:**
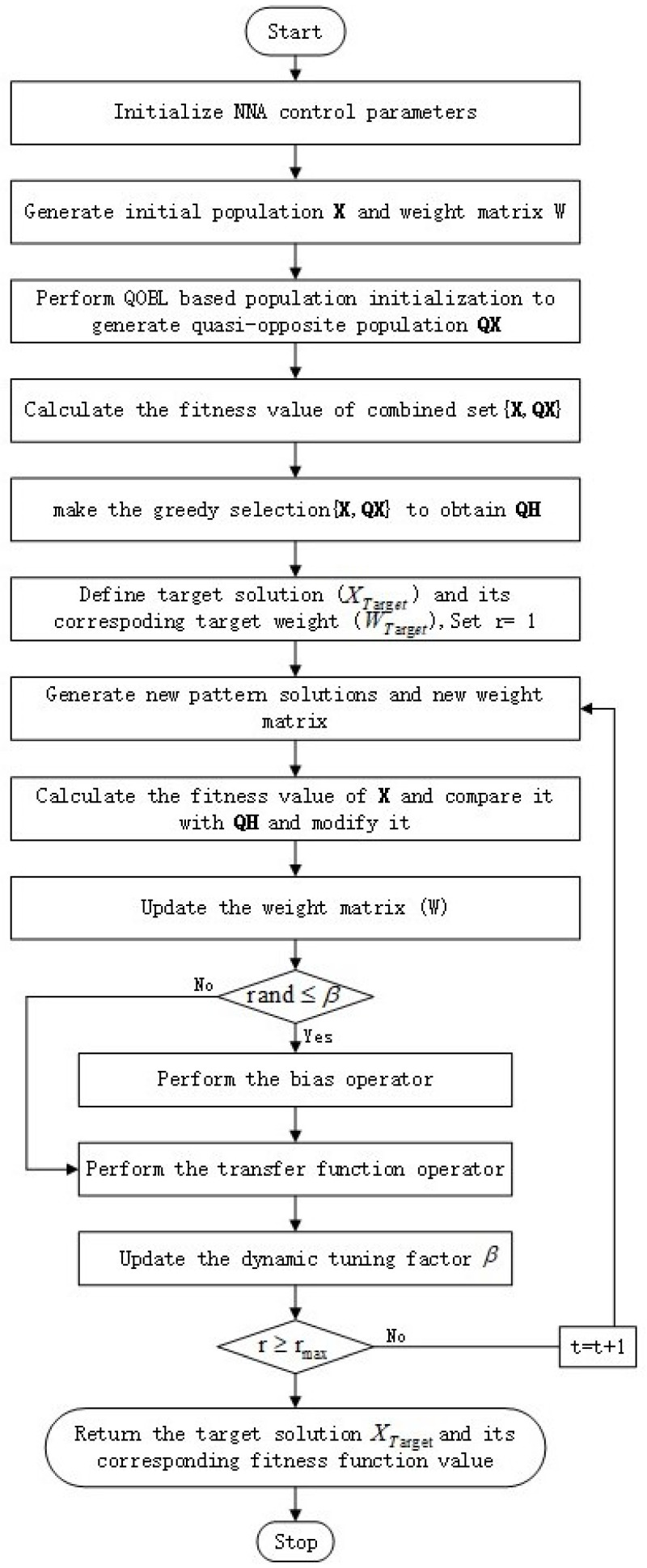
Flowchart of QOCSCNNA.

In this study, the improved algorithm by fusing the original NNA with the bias operators of QOBL, LCSC strategy, and PWLCM factor is called QOCSCNNA. The detailed flowchart as shown in Figure 3 and the pseudocode for QOCSCNNA can be found in Algorithm 4.
**Algorithm 4:** QOCSCNNA algorithmInitialize the number of iterations r (r = 1), the dynamic tuning factor $\beta^{r}(\beta^{1}=1)$Randomly generate an initial population XGenerate quasi-opposite solutions ${\tilde{QX}}_{i}$ using algorithm 2Calculate the fitness value of combined set {$X,\tilde{QX}$} then make the greedy selection to obtain $\tilde{QH}$Randomly generate the weight matrix considering the imposed constraints in Equations (1) and (2)Set the optimal solution $x_{target}^{r}$ and the optimal weight $w_{target}^{r}$While $r<r_{max}$ Generate new pattern solution $x_{i}^{r}$ by Equations (3)–(4), new weight matrix $w_{i}^{r}$ by Equation (5) Calculate the fitness value of X if $fit({\tilde{QH}}_{i})<fit(X_{i})$  $X_{i}={\tilde{QH}}_{i}$  ${fit(X}_{i})=fit(\tilde{QH})$ end if if $rand\leq \beta^{r}$  Perform the bias operator using algorithm 3 else  Perform the transfer function operator for $x_{i}^{r}$ via Equation (7) end ifCalculate the fitness value of each solution and find the optimal solution $x_{target}^{r+1}$ and the optimal weight $w_{target}^{r+1}$Update the current number of iterations by r = r + 1Update the dynamic tuning factor $\beta^{r+1}$ by Equation (17)End while

## 4. Numerical Experiments and Result Analysis

This section examines the properties of the proposed QOCSCNNA numerical optimization problems. This chapter is divided into three subsections. Section 4.1 details the CEC 2017 test function and the experimental environment that ensures the reliability of the experimental results. Section 4.2 provides a comparative analysis between QOCSCNNA and eight other metaheuristics on the CEC 2017 function which validates the effectiveness of the improved algorithms. Finally, the performance of the algorithm is compared with other algorithms through three engineering projects of practical significance in Section 4.3.

### 4.1. Experiment Setup

It is a broadly used CEC 2017 test suite [33] specifically dedicated to evaluating the performance of complex optimization algorithms. The test suite consists of 30 test functions covering a wide range of test requirements to obtain a more comprehensive insight into the performance characteristics of optimization algorithms. Unfortunately, for unavoidable reasons, the F2 test functions could not be tested, resulting in only 29 functions being tested. These functions could be categorized into four types, each with diverse levels of complexity and characteristics. Firstly, there are the single-peaked functions (F1,F3), which have a clear optimal solution and are suitable for assessing the behavior of the algorithm when dealing with simple problems. Secondly, there are simple multimodal functions (F4–F9), which have multiple partial optimal solutions and can be used to test the robustness and convergence of the algorithm during local search. The third category is hybrid functions (F11–F20), which combine the characteristics of single-peak and multimodal and are closer to the situation of complex problems in reality, enabling a comprehensive assessment of the overall global and local search capability of algorithms. Finally, the synthesized functions (F21–F30) are combined with other functions. The specific functions are shown in Table 1.

Furthermore, it was necessary to place all algorithms under the same test conditions to ensure fairness, and experiments were conducted using MATLAB R2022a software under MacOS 12.3 M1. In the CEC 2017 suite, the population size was set to 50 and the dimensionality was set to 10 D. To fully evaluate the performance of the algorithms, the maximum number of function evaluations was set to 20,000 times the population size. This setup ensures a thorough exploration of the search space, thus improving the optimization results. It is noted that the other parameters required to compare the algorithms were extracted directly from the original references to keep the consistency of the results. Moreover, there were 30 independent runs of each algorithm execution to get reliable results, and the average value (AVG) and standard deviation (STD) of the obtained results were logged.

### 4.2. QOCSCNNA for Unconstrained Benchmark Functions

To evaluate the performance of the improved algorithm, QOCSCNNA was compared with eight other well-known optimization algorithms, including NNA, CSO [36], SA [22], HHO [37], WOA [38], SCA [31], WDE [39], and RSA [40]. Based on the experimental settings outlined in Section 4.1, the average (AVG) and standard deviation (STD) of the minimum fitness values obtained on the CEC 2017 benchmark functions are presented in Table A1, with the smallest average and standard deviation highlighted in bold. When compared to other algorithms, QOCSCNNA demonstrated significant superiority in terms of both AVG and STD results in the 2017 CEC functions. Moreover, given the limited evaluation budget, the QOCSCNNA algorithm had relatively minor means and standard deviations for a range of functions including F1, F4, F5, F7, F8, F10-F17, F19-F21, F27, F29, and F30. These results highlight QOCSCNNA’s superior ability to effectively tackle optimization problems characterized by complexity and hybridity.

The results of the Wilcoxon rank-sum test (“+”, “=”, and “−” indicate that QOCSCNNA performs better, the same, or worse, respectively, compared to the other algorithms) are shown in Table A1 to better compare the performance of the different algorithms. As can be seen in the last row of Table A2, QOCSCNNA achieved significantly superior results to SA, SCA, RSA, and CSO on more than 28 test functions, while QOCSCNNA beats HHO and WOA for more than 26 functions and exceeds WDE and NNA for 23 functions. In other words, the average superiority rate of QOCSCNNA over 29 functions is 92.24% ($\sum_{i=1}^{8}\frac{{+}_{i}}{29\times 8}\times 100\%$). These results indicate that adopting the CSCL can effectively improve the optimization capability of NNA.

**Figure 4:**
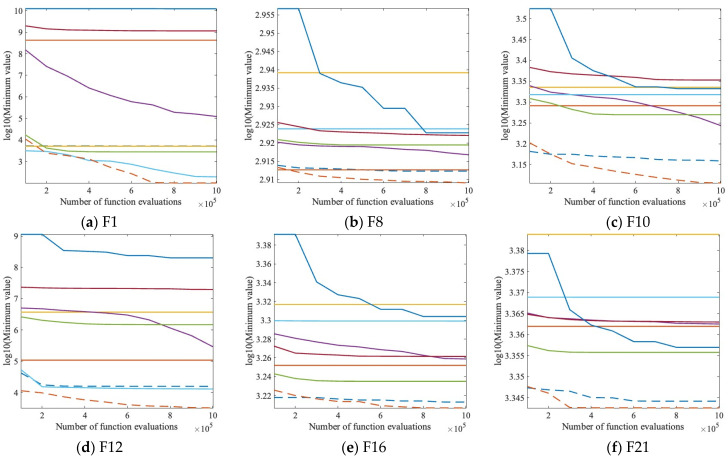

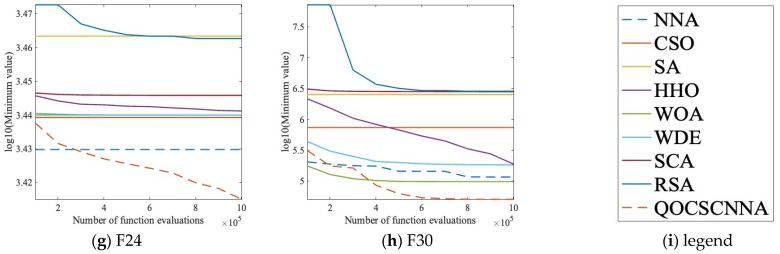
Convergence graph of QOCSC and its competitors.

Nine convergence plots of QOCSCNNA with the comparison algorithm on the CEC 2017 test set including F1, F8, F10, F12, F16, F21, F24, F29, and F30 are given in Figure 4, where the vertical axis takes the logarithm of the function’s minimum value, and the horizontal axis denotes the number of times the function was evaluated. It can be noticed that although sometimes QOCSCNNA does not perform the best in the initial phase, as the number of function iterations increases, smaller fitness values can be searched for by constantly jumping out of the local optimum. The good performance of this algorithm is because the exploration of QOBL enhances the global search capability.

### 4.3. Real-World Engineering Design Problems

Furthermore, to validate the feasibility of the QOCSCNNA for actual engineering applications, multiple algorithms were utilized to address the critical engineering design problems of cantilever beam structures (CB) [41], car side impact (CSI) [41], and tension spring (TS) [41]. For three problems, a population size of 50 was set with an iteration count of 2000 times the population size. Moreover, each algorithm was independently run 30 times to obtain reliable results. Such settings ensured thorough exploration of the search space, leading to improved optimization results. Additionally, the solution provided by QOCSCNNA was compared to well-known algorithms to better evaluate its performance.

#### 4.3.1. CB Engineering Design Problem

**Figure 5:**
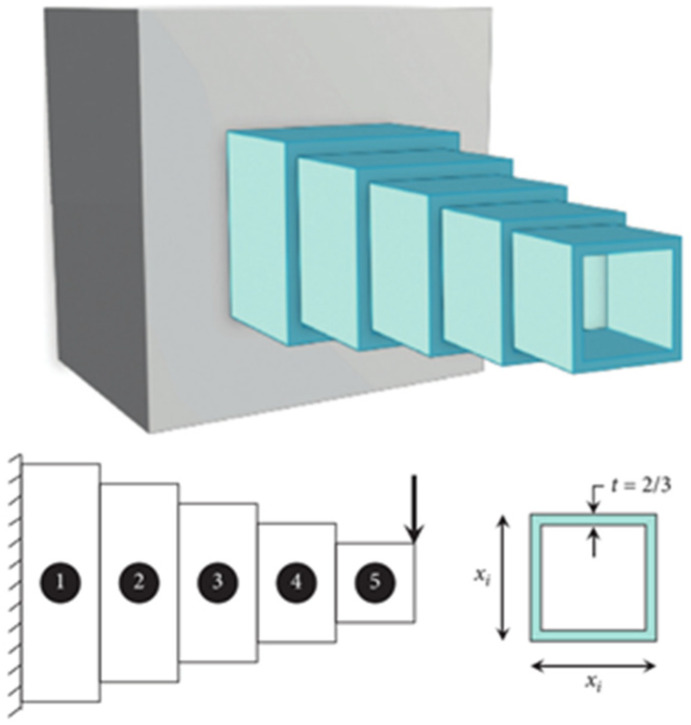
The model for the CB design problem [41].

The weight optimization of a square cross-section cantilever beam is involved in the CB structural engineering design. The beam has a rigid support at one extremity, while vertical forces act on the free nodes of the cantilever. A model of the CB design problem is illustrated in Figure 5. The beam consists of five hollow squares of equal thickness, with the height (or width) of each square being the decision variable. Meanwhile, the thickness of these squares remains constant at 2/3. The objective function of this design problem can be represented by Equation (18).

(18) $${F(x)}_{min}=0.0624\;(x_{1}+x_{2}+x_{3}+x_{4}+x_{5})$$

Subject to:

(19) $$G\left(x\right)=\frac{61}{{x_{1}}^{3}}+\frac{37}{{x_{2}}^{3}}+\frac{19}{{x_{3}}^{3}}+\frac{7}{{x_{4}}^{3}}+\frac{1}{{x_{5}}^{3}}-1\leq 0$$

Variable range:

(20) $${0.01\leq x}_{i}\leq 100,\;i=1,\;\ldots ,5.$$

This problem is being solved by several researchers using different metaheuristic methods, such as NNA, WOA, SCA, SA and PSO used in [42]. Table 2 reveals that the optimal result of QOCSCNNA is 1.3548, as well as the optimal constraints obtained from QOCSCNNA, satisfy Equation (19), which proves the validity of the optimal solutions obtained by QOCSCNNA. In addition, the optimum solutions of WOA and SA are 1.3567 and 1.3569, respectively, which are very close to the best results of QOCSCNNA. In contrast, the NNA, PSO, and SCA algorithms have poor optimum solutions, which indicates that these three algorithms are not suitable for the problem. Furthermore, by comparing the results of the Wilcoxon rank-sum test (+, =, and − indicating better, equal, or worse performance of QOCSCNNA compared to other algorithms), it is possible to discover that QOCSCNNA outperforms NNA, PSO, and SCA in terms of performance. Hence, it can be summarized that the proposed QOCSCNNA demonstrates superior feasibility compared to other algorithms.

#### 4.3.2. CSI Engineering Design Problem

**Figure 6:**
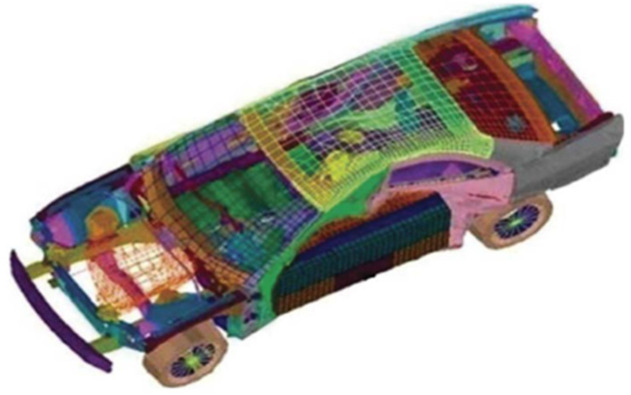
The model for the CSI problem [41].

As shown in Table 3, 11 parameters should be considered when minimizing the impact of a side impact on a vehicle. Figure 6 illustrates the model of the CSI crash design problem. The objective function of this design problem can be expressed as Equation (21):

(21) $${F\left(x\right)}_{min}=1.98+4.90x_{1}+6.67x_{2}+6.98x_{3}+4.01x_{4}+1.78x_{5}+2.73x_{7}$$

Subject to:

(22) $$G_{1}\left(x\right)=1.16-0.3717x_{2}x_{4}-0.00931x_{2}x_{10}-0.484x_{3}x_{9}+0.01343x_{6}x_{10}-1\leq 0\;$$

(23) $$G_{2}\left(x\right)=46.36-9.9x_{2}-12.9x_{1}x_{2}+0.1107x_{3}x_{10}-32\leq 0\;$$

(24) $$G_{3}\left(x\right)=33.86+2.95x_{3}+0.1792x_{3}-5.057x_{1}x_{2}-11.0x_{2}x_{8}-0.0215x_{5}x_{10}-9.98x_{7}x_{8}\;+22.0x_{8}x_{9}-32\leq 0\;$$

(25) $$G_{4}\left(x\right)=28.98+3.818x_{3}-4.2x_{1}x_{2}+0.0207x_{5}x_{10}+6.63x_{6}x_{9}-7.7x_{7}x_{8}+0.32x_{9}x_{10}-32\leq 0$$

(26) $$G_{5}\left(x\right)=0.261-0.0159x_{1}x_{2}-0.188x_{1}x_{8}-0.019x_{2}x_{7}+0.0144x_{3}x_{5}+0.0008757x_{5}x_{10}\;+0.08045x_{6}x_{9}+0.00139x_{8}x_{11}+0.00001575x_{10}x_{11}-32\leq 0$$

(27) $$G_{6}\left(x\right)=0.214+0.00817x_{5}-0.131x_{1}x_{8}-0.0704x_{1}x_{9}+0.03099x_{2}x_{6}-0.018x_{2}x_{7}+0.0208x_{3}x_{8}\;\;\;\;\;\;\;\;\;\;\;-0.02x_{2}^{2}+0.121x_{3}x_{9}-0.00364x_{5}x_{6}+0.0007715x_{5}x_{10}-0.0005354x_{6}x_{10}\;+0.00121x_{8}x_{11}+0.00184x_{9}x_{10}-0.32\leq 0$$

(28) $$G_{7}\left(x\right)=0.74-0.61x_{2}-0.163x_{3}x_{8}+0.001232x_{3}x_{10}-0.166x_{7}x_{9}+0.227x_{2}^{2}-0.32\leq 0\;$$

(29) $$G_{8}\left(x\right)=4.72-0.5x_{4}-0.19x_{2}x_{3}-0.0122x_{4}x_{10}+0.009325x_{6}x_{10}+0.000191x_{11}^{2}-4\leq 0\;$$

(30) $$G_{9}\left(x\right)=10.58-0.674x_{1}x_{2}-1.95x_{2}x_{8}+0.02054x_{3}x_{10}-0.0198x_{4}x_{10}+0.028x_{6}x_{10}-9.9\leq 0$$

(31) $$G_{10}\left(x\right)=16.45-0.489x_{3}x_{7}-0.843x_{5}x_{6}+0.0432x_{9}x_{10}-0.0556x_{9}x_{11}-0.000786x_{11}^{2}-15.7\;\leq 0\;$$

Variable range:

(32) $$0.5\leq x_{1},x_{2},x_{3},x_{4},x_{5},x_{6},x_{7}\leq 1.5\;,\;\;x_{8},x_{9}\in \left\{0.192,\;0.345\right\}\;,\;-30\leq x_{10}\;,\;x_{11}\leq +30$$

The CSI problem is a widely studied classical engineering design problem and many heuristics have been proposed to solve it over the years. The methods include NNA, SA, WOA, PSO, and SCA. According to the comparative experimental results (Table 4), the presented QOCSCNNA achieves the optimal fitness value of 23.4538 and makes the optimal constraints satisfy Equations (22)–(32). This validates the efficacy of the optimal results obtained by QOCSCNNA. In addition, the optimal results of NNA, SA, WOA, PSO, and SCA are significantly higher than those of QOCSCNNA. This indicates that QOCSCNNA holds a clear advantage among the five algorithms for solving the problem. The analysis results by the Wilcoxon rank-sum test showed that QOCSCNNA was superior to the other algorithms. It further confirms the feasibility of the obtained QOCSCNNA.

#### 4.3.3. TS Engineering Design Problem

**Figure 7:**
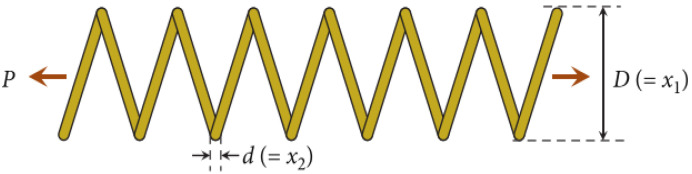
The model for the TS problem [41].

The goal of the TS problem is to reduce the weight of the spring, illustrated in Figure 7. Minimum deflection, shear stress, surge frequency, outer diameter limits, and limitations on design variables need to be considered in the design process. The parameter settings include the average coil diameter D (denoted as $x_{1}$), the wire diameter d (denoted as $x_{2}$), and the effective number of coils N (denoted as $x_{3}$). The issue is described as:

(33) $${F\left(x\right)}_{min}=(x_{3}+2)x_{2}{x_{1}}^{2}$$

Subject to:

(34) $$G_{1}\left(x\right)=1-{x_{1}}^{3}x_{2}/71785{x_{1}}^{4}\leq 0$$

(35) $$G_{2}\left(x\right)=(4{x_{2}}^{2}-x_{1}x_{2})/12566\left({x_{1}}^{3}x_{2}-{x_{1}}^{4}\right)+1/5108{x_{1}}^{2}-1\leq 0$$

(36) $$G_{3}\left(x\right)=1-{{140.45x_{1}/x}_{2}}^{2}x_{3}\leq 0$$

(37) $$G_{4}\left(x\right)={(x}_{1}+x_{2})/1.5-1\;\leq 0$$

Variable range:

(38) $$0.05\leq x_{1}\leq 2\;,\;\;0.25\leq x_{2}\leq \;1.3\;,\;2\leq x_{3}\leq 15$$

Several researchers have tried various meta-heuristics to solve this problem, including NNA, SA, WOA, PSO, and HHO. Table 5 demonstrates the optimal solutions obtained by QOCSCNNA and the comparative algorithms, and it can be seen that the proposed QOCSCNNA obtains the optimal solution, i.e., 0.127. Also, it is given that the constraints on the optimal cost achieved with QOCSCNNA meet Equations (34)–(38), which implies that the best solution provided by QOCSCNNA is valid. In addition, HHO has an optimal fitness value of 0.0129, which is nearly the same as the optimal result of QOCSCNNA. On the contrary, the optimal solutions of NNA, SA, WOA, and PSO are inferior, which means QOCSCNNA and HHO have significant advantages. Moreover, through a comparison of the results of the Wilcoxon rank-sum test, it is observed that QOCSCNNA outperforms NNA, SA, WOA, and PSO in the aspect of performance. Therefore, it can be drawn that QOCSCNNA is a more efficient and feasible method compared with other algorithms.

## 5. Conclusions and Future Works

This paper reports on the NNA based on the quasi-oppositional-based strategy, piecewise linear chaotic mapping operator, and logistic chaotic sine-cosine learning strategy proposed to enhance global search capability and convergence. More specifically, QOBL allows the generation of quasi-opposite solutions between opposite solutions and the center of the solution space during the initialization phase, helping to balance exploration and exploitation in the generation jump. A new LCSC strategy by integrating LCM and SCLS is proposed which facilitates the algorithm to control at the bias strategy stage to jump out of the local optimum. Moreover, a dynamic adjustment factor that varies with the number of evaluations is presented, which facilitates tuning the search space and accelerates the convergence speed. To demonstrate the validity of QOCSCNNA, the performance of numerical optimization problems is investigated by solving challenging CEC 2017 functions. The results of the average and standard deviation of the comparison experiments in 29 test functions show that the QOCSCNNA algorithm outperforms the NNA algorithm in 23 functions and beats the other 7 algorithms in more than half of the test functions. Meanwhile, the Wilcoxon rank-sum test and convergence analysis indicate that the QOCSCNNA algorithm significantly outperforms the other algorithms. Furthermore, QOCSCNNA and other comparative algorithms are applied to three real-world engineering design problems, and the results further evidence the applicability of the algorithms in solving practical projects.

For future research, we concentrate on the next two areas. First, QOCSCNNA will continue to be improved to address more complex real-world engineering optimal problems, which include intelligent traffic management, supply chain optimization, and large-scale unmanned aircraft systems. Second, even though QOCSCNNA can greatly enhance the global search capability of NNA, we recognize that further exploration is needed to improve the performance of NNA, especially in dealing with high-dimensional problems. Therefore, we plan to introduce the attention mechanism in neural networks for efficient exploration as well as the use of back-propagation to update the weights to further improve the performance of NNA.

## Figures and Tables

**Table 1:** The definition of the CEC2017 test suite.

	No.	Function	Optimum
Unimodal function	F1	Shifted and Rotated Bent Cigar Function	100
	F3	Shifted and Rotated Zakharov Function	300
Simple multimodal function	F4	Shifted and Rotated Rosenbrock’s Function	400
	F4	Shifted and Rotated Rastrigin’s Function	500
	F6	Shifted and Rotated Expanded Scaffer’s F6 Function	600
	F7	Shifted and Rotated Lunacek BiRastriginFunction	700
	F8	Shifted and Rotated Non-Continuous Rastrigin’s Function	800
	F9	Shifted and Rotated Levy Function	900
	F10	Shifted and Rotated Schwefel’s Function	1000
Hybrid function (HF)	F11	Hybrid Function 1 (N = 3)	1100
	F12	Hybrid Function 2 (N = 3)	1200
	F13	Hybrid Function 3 (N = 3)	1300
	F14	Hybrid Function 4 (N = 4)	1400
	F15	Hybrid Function 5 (N = 4)	1500
	F16	Hybrid Function 6 (N = 4)	1600
	F17	Hybrid Function 6 (N = 5)	1700
	F18	Hybrid Function 6 (N = 5)	1800
Hybrid function (HF)	F19	Hybrid Function 6 (N = 5)	1900
	F20	Hybrid Function 6 (N = 6)	2000
Composition function (CF)	F21	Composition Function 1 (N = 3)	2100
	F22	Composition Function 2 (N = 3)	2200
	F23	Composition Function 3 (N = 4)	2300
	F24	Composition Function 4 (N = 4)	2400
	F25	Composition Function 5 (N = 5)	2500
	F26	Composition Function 6 (N = 5)	2600
	F27	Composition Function 7 (N = 6)	2700
	F28	Composition Function 8 (N = 6)	2800
	F29	Composition Function 9 (N = 3)	2900
	F30	Composition Function 10 (N = 3)	3000
$\mathrm{Search}\;\mathrm{range}:\;{\left[-100,\;100\right]}^{D}$ (D is the population dimension)			

**Table 2:** Comparison results between QOCSCNNA and its competitor CB design problems.

	QOCSCNNA	NNA	PSO	WOA	SCA	SA
$x_{1}$	5.8487	16.0042	5.7184	5.3875	5.6072	5.7227
$x_{2}$	5.2207	4.6257	8.1930	5.7766	6.0038	6.1674
$x_{3}$	4.6131	7.0074	5.9331	4.6485	4.1554	4.3927
$x_{4}$	3.8433	2.3777	2.8983	3.7243	3.9320	3.4333
$x_{5}$	2.2120	6.6237	1.6369	2.2048	2.1331	2.0293
G(x)	−0.0258	−0.0319	−1.6287 × 10^−5^	−1.2506 × 10^−11^	−1.6002 × 10^−5^	0
${F(x)}_{min}$	**1.3564**	2.2863	1.5213	1.3567	1.3623	1.3569
+/−/=		+	+	=	+	=

**Table 3:** Influence parameters of the weight of the door.

No.	Variables	Description of Variables
1	$x_{1}$	Thicknesses of Bpillar Inner
2	$x_{2}$	Bpillar Reinforcement
3	$x_{3}$	Floor Side Inner
4	$x_{4}$	Cross Members
5	$x_{5}$	Door Beam
6	$x_{6}$	Door Beltline Reinforcement
7	$x_{7}$	Roof Rail
8	$x_{8}$	Materials of Bpillar Inner
9	$x_{9}$	Floor Side Inner
10	$x_{10}$	Barrier Height
11	$x_{11}$	Hitting Position

**Table 4:** Comparison results between QOCSCNNA and its competitor CSI design problems.

	QOCSCNNA	NNA	SA	WOA	PSO	SCA
$x_{1}$	0.5000	0.5000	0.5846	0.5676	0.5000	0.5000
$x_{2}$	1.0702	0.9389	0.8214	0.8164	0.9749	1.0438
$x_{3}$	0.5000	0.5000	0.5000	0.5282	0.5000	0.5000
$x_{4}$	1.2346	1.4497	1.3295	1.3847	1.4484	1.3642
$x_{5}$	0.5000	0.5000	0.5386	0.5000	0.5000	0.5000
$x_{6}$	1.3072	0.7040	1.2600	0.6988	1.5000	0.9223
$x_{7}$	0.9359	1.1196	1.3245	1.2616	1.1604	0.9757
$x_{8}$	0.9200	0.9200	0.9200	0.9200	0.9200	0.9200
$x_{9}$	0.9200	0.9200	0.9200	0.9200	0.9200	0.9200
$x_{10}$	1.5200	0.5787	−0.6113	−5.1295	−26.1752	−7.1364
$x_{11}$	1.2847	15.7543	10.0525	2.1609	1.4852	−1.9988
$G_{1}(x)$	−0.5422	−0.5681	−0.4742	−0.5046	−0.8772	−0.6109
$G_{2}(x)$	−3.0534	−0.9586	0	−3.2758 × 10^−4^	−3.0287	−3.1005
$G_{3}(x)$	−0.1003	−0.1158	−0.8491	−3.9851 × 10^−9^	−0.6587	−0.0384
$G_{4}(x)$	−1.5518	−6.5431	−5.0120	−9.1875	−10.2060	−6.7642
$G_{5}(x)$	−0.0703	−0.0967	−0.0787	−0.1282	−0.0703	−0.1067
$G_{6}(x)$	−0.1258	−0.1282	−0.1378	−0.1600	−0.1578	−0.1548
$G_{7}(x)$	−0.1898	−0.1982	−0.2055	−0.2019	−0.2273	−0.1978
$G_{8}(x)$	−0.0030	−0.0531	−7.3880 × 10^−4^	−1.6522 × 10^−4^	−2.5206 × 10^−9^	−0.0031
$G_{9}(x)$	−1.5665	−1.3500	−1.1290	−1.1124	−2.0150	−1.6091
$G_{10}(x)$	−0.0364	−0.7984	−0.7851	−0.1884	−1.2840	−0.0618
${F(x)}_{min}$	**23.4538**	23.9420	23.7545	23.7803	24.2888	23.9060
+/−/=		+	+	+	+	+

**Table 5:** Comparison results between QOCSCNNA and its competitor TS design problems.

	QOCSCNNA	NNA	SA	WOA	PSO	HHO
$x_{1}$	0.5028	0.0659	0.0556	0.0612	0.0649	0.0552
$x_{2}$	0.3850	0.8040	0.4592	0.6309	0.7659	0.4478
$x_{3}$	9.8066	5.2301	10.0143	4.0040	6.8458	7.4345
$G_{1}(x)$	−1.1266 × 10^−10^	−1.0099	−0.4100	−2.7309 × 10^−6^	−1.4141	−1.3636 × 10^−8^
$G_{2}(x)$	−1.1102 × 10^−16^	−1.2845 × 10^−11^	−1.1266 × 10^−7^	−1.4795 × 10^−10^	−5.3960 × 10^−6^	−3.3307 × 10^−16^
$G_{3}(x)$	−490.0553	−73.8673	−370.1311	−85.4248	−105.3872	−286.5735
$G_{4}(x)$	−0.7081	−0.4201	−0.6568	−0.5386	−0.4461	−0.6647
${F(x)}_{min}$	**0.0127**	0.0252	0.0171	0.0142	0.0285	0.0129
+/−/=		+	+	+	+	=

## Data Availability

Not applicable.

## Appendix A

**Table A1:** Comparison results of algorithms on CEC 2017.

No.	Metric	QOCSCNNA	NNA	CSO	SA	HHO	WOA	WDE	SCA	RSA
F1	AVG	**1.0000 × 10^3^**	5.2273 × 10^3^	4.2623 × 10^8^	5.0774 × 10^3^	1.2246 × 10^5^	2.7875 × 10^3^	1.9213 × 10^2^	1.1539 × 10^9^	1.2285 × 10^10^
	STD	**1.5594 × 10^−4^**	4.2122 × 10^3^	8.0384 × 10^8^	3.5549 × 10^3^	5.5971 × 10^4^	2.7429 × 10^3^	2.1334 × 10^2^	1.6023 × 10^9^	3.6113 × 10^9^
F3	AVG	3.0000 × 10^2^	3.0000 × 10^2^	4.1083 × 10^3^	3.1815 × 10^4^	3.0031 × 10^2^	3.0016 × 10^2^	**3.0000 × 10^2^**	3.4470 × 10^3^	9.3828 × 10^3^
	STD	8.9486 × 10^−12^	1.0010 × 10^−9^	2.8705 × 10^3^	1.5764 × 10^4^	1.4204 × 10^−1^	1.7345 × 10^−1^	**5.2034 × 10^−13^**	2.9124 × 10^3^	4.6166 × 10^3^
F4	AVG	**4.0006 × 10^2^**	4.0199 × 10^2^	4.3755 × 10^2^	4.2943 × 10^2^	4.0792 × 10^2^	4.0945 × 10^2^	4.0533 × 10^2^	4.8410 × 10^2^	1.5297 × 10^3^
	STD	**3.4750 × 10^−1^**	1.0132 × 10^1^	3.1247 × 10^1^	3.3827 × 10^1^	1.6671 × 10^1^	1.6874 × 10^1^	1.7456 × 10^1^	6.1224 × 10^1^	5.9168 × 10^2^
F5	AVG	**5.1149 × 10^2^**	5.1761 × 10^2^	5.2463 × 10^2^	5.7441 × 10^2^	5.3595 × 10^2^	5.4435 × 10^2^	5.4026 × 10^2^	5.4776 × 10^2^	5.8814 × 10^2^
	STD	**4.4021 × 10^0^**	6.4411 × 10^0^	6.7348 × 10^0^	3.3369 × 10^1^	1.4968 × 10^1^	1.2415 × 10^1^	1.8390 × 10^1^	1.5013 × 10^1^	2.6776 × 10^1^
F6	AVG	6.0039 × 10^2^	**6.0000 × 10^2^**	6.1112 × 10^2^	6.6755 × 10^2^	6.1670 × 10^2^	6.1923 × 10^2^	6.2053 × 10^2^	6.2147 × 10^2^	6.3924 × 10^2^
	STD	2.5611 × 10^−1^	**1.3743 × 10^−6^**	7.7939 × 10^0^	2.3173 × 10^1^	1.0198 × 10^1^	8.3311 × 10^0^	1.3073 × 10^1^	9.2855 × 10^0^	1.1581 × 10^1^
F7	AVG	**7.2388 × 10^2^**	7.2719 × 10^2^	7.3238 × 10^2^	7.9634 × 10^2^	7.6245 × 10^2^	7.6965 × 10^2^	7.7299 × 10^2^	7.8204 × 10^2^	8.1741 × 10^2^
	STD	**6.6604 × 10^0^**	6.3264 × 10^0^	1.1115 × 10^1^	5.3283 × 10^1^	1.6185 × 10^1^	1.7812 × 10^1^	2.5893 × 10^1^	2.1705 × 10^1^	4.2648 × 10^1^
F8	AVG	**8.1108 × 10^2^**	8.1716 × 10^2^	8.1781 × 10^2^	8.6931 × 10^2^	8.2558 × 10^2^	8.3072 × 10^2^	8.3917 × 10^2^	8.3569 × 10^2^	8.3708 × 10^2^
	STD	**4.9211 × 10^0^**	6.7785 × 10^0^	5.4251 × 10^0^	2.6543 × 10^1^	6.0887 × 10^0^	1.3195 × 10^1^	1.7063 × 10^1^	9.8691 × 10^0^	1.1248 × 10^1^
F9	AVG	9.0257 × 10^2^	**9.0002 × 10^2^**	9.4739 × 10^2^	3.0775 × 10^3^	1.1992 × 10^3^	1.1020 × 10^3^	1.7433 × 10^3^	1.2542 × 10^3^	1.5024 × 10^3^
	STD	2.5656 × 10^0^	**8.4907 × 10^−2^**	3.8012 × 10^1^	1.7799 × 10^3^	2.5726 × 10^2^	1.9554 × 10^2^	5.9984 × 10^2^	2.2817 × 10^2^	3.9959 × 10^2^
F10	AVG	**1.2767 × 10^3^**	1.4417 × 10^3^	1.9552 × 10^3^	2.1637 × 10^3^	1.7521 × 10^3^	1.8604 × 10^3^	2.0783 × 10^3^	2.2525 × 10^3^	2.1479 × 10^3^
	STD	**1.7181 × 10^2^**	2.3379 × 10^2^	2.9479 × 10^2^	3.7853 × 10^2^	2.2240 × 10^2^	2.5633 × 10^2^	3.8612 × 10^2^	3.5089 × 10^2^	3.0579 × 10^2^
F11	AVG	**1.1080 × 10^3^**	1.1152 × 10^3^	1.1726 × 10^3^	1.2969 × 10^3^	1.1380 × 10^3^	1.1501 × 10^3^	1.1149 × 10^3^	1.5303 × 10^3^	4.3697 × 10^3^
	STD	**4.8079 × 10^0^**	9.1784 × 10^0^	4.9546 × 10^1^	2.5319 × 10^2^	3.7571 × 10^1^	4.8840 × 10^1^	8.7095 × 10^0^	6.7499 × 10^2^	1.7531 × 10^3^
F12	AVG	**3.2398 × 10^3^**	1.5886 × 10^4^	1.0761 × 10^5^	3.7010 × 10^6^	2.9019 × 10^5^	1.4733 × 10^6^	1.3037 × 10^4^	1.9745 × 10^7^	2.0259 × 10^8^
	STD	**3.0675 × 10^3^**	1.5987 × 10^4^	2.2332 × 10^5^	3.5550 × 10^6^	3.2864 × 10^5^	1.9770 × 10^6^	1.2535 × 10^4^	7.6865 × 10^7^	4.5420 × 10^8^
F13	AVG	**1.3145 × 10^3^**	7.5368 × 10^3^	3.5358 × 10^3^	1.8885 × 10^4^	1.3338 × 10^4^	1.5718 × 10^4^	1.3429 × 10^3^	1.3325 × 10^4^	1.6636 × 10^7^
	STD	**6.6758 × 10^0^**	5.9487 × 10^3^	2.2158 × 10^3^	1.1688 × 10^4^	9.5387 × 10^3^	1.1828 × 10^4^	5.0494 × 10^1^	6.8891 × 10^3^	2.9435 × 10^7^
F14	AVG	**1.4061 × 10^3^**	1.4256 × 10^3^	1.4532 × 10^3^	1.3088 × 10^4^	1.4963 × 10^3^	1.4910 × 10^3^	1.4494 × 10^3^	1.5214 × 10^3^	3.2583 × 10^3^
	STD	**3.2039 × 10^0^**	1.2052 × 10^1^	1.9130 × 10^1^	1.0440 × 10^4^	1.9157 × 10^1^	2.8546 × 10^1^	8.1283 × 10^1^	5.0084 × 10^1^	1.1504 × 10^3^
F15	AVG	**1.5043 × 10^3^**	1.5078 × 10^3^	2.0383 × 10^3^	1.2101 × 10^4^	1.5800 × 10^3^	1.7077 × 10^3^	1.5124 × 10^3^	6.3238 × 10^3^	4.2151 × 10^3^
	STD	**2.6053 × 10^0^**	5.6853 × 10^0^	1.5397 × 10^3^	9.2005 × 10^3^	5.0374 × 10^1^	9.6909 × 10^1^	7.7796 × 10^0^	4.2253 × 10^3^	2.2369 × 10^3^
F16	AVG	**1.6101 × 10^3^**	1.6333 × 10^3^	1.7866 × 10^3^	2.0739 × 10^3^	1.8147 × 10^3^	1.7182 × 10^3^	1.9915 × 10^3^	1.8258 × 10^3^	2.0139 × 10^3^
	STD	**2.4942 × 10^1^**	4.8432 × 10^1^	1.2516 × 10^2^	2.1413 × 10^2^	1.4412 × 10^2^	1.0270 × 10^2^	1.9766 × 10^2^	1.2447 × 10^2^	2.0499 × 10^2^
F17	AVG	**1.7081 × 10^3^**	1.7218 × 10^3^	1.7547 × 10^3^	1.8582 × 10^3^	1.7640 × 10^3^	1.7726 × 10^3^	1.8639 × 10^3^	1.7741 × 10^3^	1.8074 × 10^3^
	STD	**8.2533 × 10^0^**	1.9107 × 10^1^	1.6551 × 10^1^	1.0271 × 10^2^	3.1636 × 10^1^	3.7196 × 10^1^	1.1954 × 10^2^	2.6842 × 10^1^	4.9826 × 10^1^
F18	AVG	2.2765 × 10^3^	1.3327 × 10^4^	2.8110 × 10^3^	2.2214 × 10^4^	1.4777 × 10^4^	1.6149 × 10^4^	**1.8208 × 10^3^**	1.6568 × 10^4^	1.3899 × 10^7^
	STD	9.9498 × 10^2^	1.0439 × 10^4^	2.0918 × 10^3^	1.4389 × 10^4^	1.0784 × 10^4^	1.1130 × 10^4^	**9.2447 × 10^0^**	1.3148 × 10^4^	4.7873 × 10^7^
F19	AVG	**1.9014 × 10^3^**	1.9021 × 10^3^	2.0291 × 10^3^	9.9946 × 10^3^	6.0167 × 10^3^	9.8421 × 10^3^	1.9094 × 10^3^	1.9580 × 10^4^	2.0567 × 10^5^
	STD	**5.6987 × 10^−1^**	2.8074 × 10^0^	3.8966 × 10^2^	1.1165 × 10^4^	4.8818 × 10^3^	7.7129 × 10^3^	5.9387 × 10^0^	4.6590 × 10^4^	3.5967 × 10^5^
F20	AVG	**2.0042 × 10^3^**	2.0178 × 10^3^	2.0765 × 10^3^	2.2958 × 10^3^	2.1063 × 10^3^	2.0754 × 10^3^	2.1583 × 10^3^	2.1302 × 10^3^	2.1900 × 10^3^
	STD	**5.2200 × 10^0^**	1.1072 × 10^1^	4.1266 × 10^1^	1.2813 × 10^2^	6.1903 × 10^1^	3.4860 × 10^1^	9.0067 × 10^1^	6.2912 × 10^1^	6.6794 × 10^1^
F21	AVG	**2.2009 × 10^3^**	2.2090 × 10^3^	2.3012 × 10^3^	2.4203 × 10^3^	2.3042 × 10^3^	2.2687 × 10^3^	2.3383 × 10^3^	2.3068 × 10^3^	2.2749 × 10^3^
	STD	**1.1942 × 10^0^**	2.7408 × 10^1^	4.2005 × 10^1^	5.2922 × 10^1^	5.9704 × 10^1^	6.7893 × 10^1^	3.2279 × 10^1^	5.5607 × 10^1^	5.2630 × 10^1^
F22	AVG	2.3084 × 10^3^	**2.2965 × 10^3^**	2.3221 × 10^3^	3.1894 × 10^3^	2.3131 × 10^3^	2.3356 × 10^3^	2.6411 × 10^3^	2.3760 × 10^3^	3.0777 × 10^3^
	STD	1.9126 × 10^1^	1.9511 × 10^1^	2.0456 × 10^1^	7.4665 × 10^2^	**5.3597 × 10^0^**	1.8075 × 10^2^	5.6473 × 10^2^	1.2571 × 10^2^	3.6595 × 10^2^
F23	AVG	**2.6181 × 10^3^**	2.6248 × 10^3^	2.6322 × 10^3^	2.8207 × 10^3^	2.6505 × 10^3^	2.6385 × 10^3^	2.6384 × 10^3^	2.6448 × 10^3^	2.6855 × 10^3^
	STD	7.8347 × 10^0^	**7.3500 × 10^0^**	1.8049 × 10^1^	1.9624 × 10^2^	2.0815 × 10^1^	1.4541 × 10^1^	2.3021 × 10^1^	2.5283 × 10^1^	1.7166 × 10^1^
F24	AVG	**2.6003 × 10^3^**	2.6901 × 10^3^	2.7495 × 10^3^	2.9065 × 10^3^	2.7617 × 10^3^	2.7542 × 10^3^	2.7540 × 10^3^	2.7913 × 10^3^	2.9018 × 10^3^
	STD	1.2995 × 10^2^	1.1722 × 10^2^	**3.9062 × 10^1^**	8.9372 × 10^1^	9.2439 × 10^1^	7.1181 × 10^1^	7.1111 × 10^1^	6.0177 × 10^1^	8.8390 × 10^1^
F25	AVG	**2.9134 × 10^3^**	2.9334 × 10^3^	2.9497 × 10^3^	2.9915 × 10^3^	2.9278 × 10^3^	2.9324 × 10^3^	2.9303 × 10^3^	2.9939 × 10^3^	3.4258 × 10^3^
	STD	2.1652 × 10^1^	2.2886 × 10^1^	**2.0807 × 10^1^**	9.7024 × 10^1^	2.3714 × 10^1^	2.4213 × 10^1^	2.4698 × 10^1^	8.0498 × 10^1^	1.9369 × 10^2^
F26	AVG	2.9770 × 10^3^	**2.9559 × 10^3^**	3.1403 × 10^3^	4.0366 × 10^3^	3.2685 × 10^3^	3.1671 × 10^3^	3.5374 × 10^3^	3.3500 × 10^3^	4.1195 × 10^3^
	STD	8.3544 × 10^1^	**4.3782 × 10^1^**	1.5730 × 10^2^	6.8739 × 10^2^	5.2972 × 10^2^	3.0177 × 10^2^	6.1491 × 10^2^	3.3099 × 10^2^	3.4946 × 10^2^
F27	AVG	**3.0921 × 10^3^**	3.0932 × 10^3^	3.1089 × 10^3^	3.2479 × 10^3^	3.1213 × 10^3^	3.1193 × 10^3^	3.1127 × 10^3^	3.1621 × 10^3^	3.2831 × 10^3^
	STD	**1.9594 × 10^0^**	2.8759 × 10^0^	1.3637 × 10^1^	6.4546 × 10^1^	2.4699 × 10^1^	3.2729 × 10^1^	1.9635 × 10^1^	3.8743 × 10^1^	7.4340 × 10^1^
F28	AVG	3.3480 × 10^3^	**3.2893 × 10^3^**	3.3576 × 10^3^	3.6733 × 10^3^	3.3046 × 10^3^	3.3119 × 10^3^	3.3573 × 10^3^	3.5270 × 10^3^	3.5508 × 10^3^
	STD	1.0843 × 10^2^	**8.3349 × 10^1^**	1.3117 × 10^2^	1.9834 × 10^2^	1.5539 × 10^2^	1.5543 × 10^2^	1.1627 × 10^2^	1.9909 × 10^2^	1.0554 × 10^2^
F29	AVG	**3.1627 × 10^3^**	3.1851 × 10^3^	3.2150 × 10^3^	3.4427 × 10^3^	3.2798 × 10^3^	3.2644 × 10^3^	3.3194 × 10^3^	3.2993 × 10^3^	3.4197 × 10^3^
	STD	**2.0458 × 10^1^**	3.4660 × 10^1^	3.7003 × 10^1^	1.5928 × 10^2^	7.1812 × 10^1^	6.0127 × 10^1^	7.5910 × 10^1^	7.9825 × 10^1^	1.5867 × 10^2^
F30	AVG	**5.0572 × 10^4^**	1.1594 × 10^5^	7.3684 × 10^5^	2.5244 × 10^6^	1.8827 × 10^5^	9.7804 × 10^4^	1.8197 × 10^5^	2.8045 × 10^6^	2.8547 × 10^6^
	STD	**1.5162 × 10^5^**	2.8149 × 10^5^	1.1491 × 10^6^	2.6234 × 10^6^	3.9800 × 10^5^	1.6309 × 10^5^	3.6619 × 10^5^	6.4617 × 10^6^	6.5786 × 10^6^

**Table A2:** The Wilcoxon rank-sum test results.

No.	NNA	CSO	SA	HHO	WOA	WDE	SCA	RSA
F1	+	+	+	+	+	+	+	+
F3	+	+	+	+	+	=	+	+
F4	+	+	+	+	+	+	+	+
F5	+	+	+	+	+	+	+	+
F6	−	+	+	+	+	+	+	+
F7	+	+	+	+	+	+	+	+
F8	+	+	+	+	+	+	+	+
F9	−	+	+	+	+	+	+	+
F10	+	+	+	+	+	+	+	+
F11	+	+	+	+	+	+	+	+
F12	+	+	+	+	+	+	+	+
F13	+	+	+	+	+	+	+	+
F14	+	+	+	+	+	+	+	+
F15	+	+	+	+	+	+	+	+
F16	+	+	+	+	+	+	+	+
F17	+	+	+	+	+	+	+	+
F18	+	+	+	+	+	=	+	+
F19	+	+	+	+	+	+	+	+
F20	+	+	+	+	+	+	+	+
F21	+	+	+	+	+	+	+	+
F22	−	+	+	=	+	=	+	+
F23	+	+	+	+	=	+	+	+
F24	+	+	+	+	+	+	+	+
F25	+	+	+	=	+	=	+	+
F26	=	+	+	+	+	+	+	+
F27	=	+	+	+	+	+	+	+
F28	−	=	+	=	−	=	+	+
F29	+	+	+	+	+	+	+	+
F30	+	+	+	+	+	=	+	+
Statistics Number (+/−/=)	23/4/2	28/0/1	29/0/0	26/0/3	27/1/1	23/0/6	29/0/0	29/0/0
